# Young Adults' Views on Priority Health Issues and Their Involvement in Shaping Responses: A Qualitative Exploration in South Australia

**DOI:** 10.1002/hpja.70202

**Published:** 2026-06-05

**Authors:** Patience Castleton, Zahra Ali Padhani, Jaameeta Kurji, Stuart Vass, Sara Ataie, Salima Meherali, Zohra S. Lassi

**Affiliations:** ^1^ School of Public Health, College of Health Adelaide University Adelaide South Australia Australia; ^2^ Robinson Research Institute, Adelaide University Adelaide South Australia Australia; ^3^ Young Adult Health & Wellbeing, the Kids Research Institute Australia Adelaide South Australia Australia; ^4^ National Centre for Epidemiology and Population Health, Australian National University Canberra Australia; ^5^ School of Psychology, Adelaide University Adelaide South Australia Australia; ^6^ School of Computer and Mathematical Sciences, Adelaide University Adelaide South Australia Australia; ^7^ College of Medicine and Public Health, Flinders University Adelaide South Australia Australia; ^8^ College of Health Sciences, Faculty of Nursing, University of Alberta Edmonton Canada

**Keywords:** co‐design, consumer involvement, health priorities, young adults

## Abstract

**Introduction:**

Young people must be central to health response design to ensure they are relevant and effective. It requires a clearer understanding of the opportunities and expectations surrounding youth engagement. Therefore, this study aims to gain a deeper understanding of the health priorities of young adults residing in South Australia (SA) and their engagement in the design of health programmes.

**Methods:**

In‐depth interviews were conducted with 13 young adults aged 18–24 years (all genders) in SA to identify priority health concerns, contributing factors and preferred solutions. We also explored their interest and expectations of being involved in the health programme design. Thematic analysis was conducted.

**Results:**

Mental health and vaping emerged as the predominant health concerns. Key drivers of health concern amongst young adults included a lack of awareness of vaping and eating disorders, peer pressure, living costs, poor work‐life balance and limited mental health services. Participants recommended early, school‐based interventions, and free accessible services. They underscored the need for structural changes at policy‐level to create supportive environments. Participants expressed interest in contributing to intervention design when aligned with their interests, skills and availability, but reported limited opportunities.

**Conclusion:**

Young adults are eager to be involved in health programme design and have unique skills that should be better leveraged upon through co‐design of research and programmes. Effective solutions to pressing health concerns must align with the lives of young adults and actively involve them in the design, implementation and evaluation of programmes and policies.

**What's Next?:**

The study results suggest employing the motivation and insights of young adults can lead to more relevant, effective and engaging health solutions tailored to their needs.

## 1 Introduction

Adolescence is a period of rapid physical, neurological, emotional and psychosocial transformation in an individual's life. It presents a valuable second opportunity to consolidate the foundations of good health after the first 1000 days of life [1]. Investment in young adult health and wellbeing has the potential to yield significant ‘triple dividends of benefits’: improve their current health, positively shape trajectories into adulthood and influence the well‐being of future generations [2]. In Australia, mental health conditions and substance use disorders are key contributors to the burden of disease in older young adults [3]. The 2020–2022 National Study of Mental Health and Wellbeing (NSMHWB) reported very high levels of psychological distress amongst young people aged 16–24 years [4], with the 2021 Household, Income and Labour Dynamics in Australia (HILDA) survey similarly reporting high psychological distress (42.3%) and loneliness amongst young people aged 15–24 years [5]. Additionally, in the 2024 Youth Survey, 35% of young people voice concerns about high levels of vaping amongst adolescents in Australia [6]. Young Australians are also struggling to deal with the ongoing cost of living crisis, with 27% of participants in The Advocate for Children and Young People 2023 survey mentioning they could not afford to seek medical services even when unwell. This wide range of mental and physical health concerns faced by young Australians urges the need for health promotion interventions to be better tailored for their young audience, thus promoting the need for co‐designed research and health programmes.

Consumer‐driven research was recently listed as the top priority in the ‘Australian Medical Research and Innovation Priorities 2022–2024’ [7]. It acknowledges that incorporating consumers' needs and experiences in research allows for outcomes to be better ‘fit‐for‐purpose’ [7]. The engagement of young adults in all aspects of activities designed for their personal and community development, including their empowerment to contribute to decisions about their personal, family, social, economic and political development, has been recognised by the United Nations as their fundamental right [8]. Whilst small efforts to increase the engagement of young adults in co‐designing programmes have begun in Australia, it is not well understood how we should be ensuring active, purposeful engagement should be conducted with young people at the forefront [9]. Young respondents in the recent 2024 SA Youth Action Plan, involving over 800 young people in workshops and surveys, emphasised their desire to be better involved in designing and implementing the policies that affect them, expressing frustration when their views are not considered [10]. However, this plan did not further investigate how to engage these young people meaningfully in co‐designing programmes and services. Thus, many current efforts to engage them in co‐design may fall short of meeting their unique needs and should be prioritised in research.

Meaningful engagement in co‐designing programmes means that individuals have the opportunity to participate in all stages of a project, including planning, action, observation and reflection [11, 12]. However, it is important to acknowledge the barriers and struggles of co‐designing programmes and youth engagement. For example, financial barriers with regard to reimbursements may present a significant barrier to ethically engaging young adults in programmes [13]. Further, concerns around the abilities of young adults to cope with the demands of involvement, lack of understanding in health programmes and boredom in sustainable contribution have been previously raised [13]. Therefore, to create appropriate and engaging health programmes for Australian youth and enhance youth involvement in programme designing, it is important to better understand exactly how young adults want to be involved in co‐designing programmes.

It is also important that we understand the health priorities and needs of adolescents and young adults directly from them, thus allowing us to co‐design interventions with their most pressing needs first. Seeking young adults' views on what the most neglected aspects of young people's health care are will allow us to develop a deep understanding of what interventions and programmes will be of greatest benefit to them, thus ensuring the greatest benefit and engagement in co‐designing programmes [10, 14].

Engaging young adults in co‐designing health interventions and programmes is vital in ensuring the widespread adoption of these programmes with far‐reaching accessibility and usability. Soliciting input from direct consumers leads to increased engagement with health programmes and contributes to improved health outcomes for young adults, underscoring the importance of their direct involvement in research programmes. Therefore, this study aimed to gain a deeper understanding of the health priorities of young adults (18 to 24 years) residing in SA and their engagement in the design of health programmes.

## 2 Methods

To address the objective, we undertook a qualitative exploratory study adhering to the Standards for Reporting Qualitative Research checklist for reporting results [15] (File S1). We conducted in‐depth interviews with adolescents and young adults residing in Adelaide, South Australia. This study was conducted between July 2023 and May 2024, with all interviews completed by the end of 2023. A South Australia‐based youth advisory group was established in March 2023 by Z.S.L. at the University of Adelaide (now Adelaide University), for a separate research study, consisting of five young adults. The advisory group members were invited to provide input around key study activities, including recruitment, data collection and data interpretation. Two members (S.A. and S.V.) expressed interest and played key roles in all aspects of the study, including assisting in snowball sampling recruitment, initial identification of interview questions, reviewing data summaries to agree on key themes and manuscript drafting. The advisory group members also reviewed and provided inputs to the final manuscript.

### 2.1 Setting and Study Participants

The study was conducted in Adelaide, South Australia and included 13 adolescents and young adults (18–24 years). Study participants were identified through professional networks, organisations providing health services to young people and tertiary education institutions. Participants were recruited through distribution and placement of flyers at the Adelaide University and public libraries. They were also recruited by posting flyers on different social media platforms such as LinkedIn, X, and Instagram. Snowball sampling was also employed, where participants made referrals. Those expressing interest in participating were contacted via email by the research team.

### 2.2 Data Collection and Management

A semi‐structured interview guide with prompts was followed for conducting interviews with study participants to explore what young adults felt were their most pressing health concerns and recommendations to effectively and appropriately address their needs. Interest in active roles in shaping responses through research or programmatic design was also specifically explored. Question guidelines can be found in Supporting Information S2. Interviews were conducted in English, online or in person, depending on participant preferences. They typically lasted about 30 min and were conducted by trained researchers, P.C. (from Australia) and ZAP (from Pakistan), who are female doctoral students. P.C. is also a young adult. Interviews were audio‐recorded with participant consent for transcription purposes. The transcript generated via Zoom was reviewed for accuracy (P.C.); notes were taken if a participant declined audio recording. Based on the scope of the investigation, we estimated the number of participants required to obtain a reasonable sense of the priorities and finalised sample recruitment once data saturation was achieved. Assistance from the advisory group members aided in determining when recruitment should be ceased.

### 2.3 Analysis

De‐identified transcripts were then analysed in dual by P.C. and Z.A.P. using thematic analysis to identify patterns across cases (i.e., young adults) and to distil key ideas. These were discussed with the rest of the author team and two advisory group members (S.A. and S.V.), who expressed an interest in the process. The decision to use thematic analysis was guided by its ‘theoretical flexibility’ [16]. A ‘codebook approach’ was used, where codes (key ideas represented in short segments of text) were used to map the analytic process following a constructivist paradigm. Rather than relying on a codebook to assess reliability and to standardise coding across coders, the codebook served as a point of discussion to chart emerging patterns during the analysis process. This approach best represents the theoretical learnings of the research team, who acknowledge that meaning is socially constructed through interaction between individuals. Analysis was performed in NVIVO software (2024/version 14) [17].

### 2.4 Author Reflexivity

We acknowledge that our individual life experiences, worldviews and knowledge systems shape our understanding of issues and what meaning we make of the data. To recognise this, we approached analysis as a product of our interaction with the data. Through our discussions, we explicitly examined how our own experiences and backgrounds may have influenced what stood out to us in the data. We tried to assemble a group of diverse individuals (researchers and youth advisors) across various ages, cultural and social identities, lived experience and research experience. The first author (P.C.) and lead interviewer was a young person herself (< 25 years), with experience in co‐designing research with young adults, which facilitated rapport and helped foster a comfortable and relatable interview environment for participants. The research team includes a mix of qualitative and quantitative researchers who have post‐positivist and constructivist leanings, are committed to social justice and tackling ageism and have personal motivations for advancing equity. Whilst we have included a positionality statement, we agree with Savolainen and colleagues that holding ourselves accountable to the integrity of the ‘collective process of truth‐seeking’ carries far more weight [18].

## 3 Results

This study included face‐to‐face interviews with 13 young adults aged between 18 and 24 years old, five of whom were recruited through snowball sampling. The majority of participants were young women (*n* = 11), and there was roughly an even split between young adults born in Australia (*n* = 7) and those who had migrated from other countries (such as Central and South Asian and European countries). Two participants identified as members of the queer community. Most participants were enrolled in tertiary education institutions or had completed tertiary studies (*n* = 9). Most participants still lived in their family homes with their parents or other family members (*n* = 8). All participants were working part‐time or full‐time in addition to being enrolled in an educational institution. Two participants declined audio recording; thus, handwritten notes were taken during the interview.

### 3.1 Key Health and Well‐Being Concerns and Drivers

Mental health emerged as a predominant concern amongst all participants. Depression and anxiety were specifically mentioned across all interviews, whilst some listed eating disorders and post‐traumatic stress disorder. During interviews, participants unpacked what they thought was driving these issues, often highlighting the interrelated nature of issues and the multiple levels at which determinants operated. The extracts below illustrate the dynamics between peer relationships, ‘lifestyle choices’ and health.

There's a whole stigma about trying to fit in and do what other people are doing, and that often includes activities such as sex and drugs, and alcohol, especially at the younger ages. Yeah, vaping now and in the younger ages like early, early teens, even earlier is crazy. *(Participant # 5)*.

When it comes to stuff like peer pressure situations or likes at parties, you feel at least a bit more forced to do certain things that you might not normally do… I do believe that there is just a whole stigmatism about trying to fit in and do what other people are doing. *(Participant # 6)*.

Maintaining balance between education, work commitments and personal life was believed to be a significant factor in preserving mental well‐being; however, system‐level factors often contributed to increased anxiety levels. For example, some participants expressed concerns that the education system is not optimally structured to support students with part‐time jobs. Excessive workloads and tight schedules made it difficult to balance studies with employment, which many young students considered essential due to the financial crisis. Additionally, schools and universities were reported to have insufficient mental health support for students who were struggling to manage their studies with their personal lives.

Everyone I know is overworked, over‐stressed and anxious about their future and the future progression of their financial status… Schools and Universities need to plan workloads a bit better to accommodate working lifestyles as well. Just better planning and respect for the students' timetables and workloads. *(Participant # 2)*.

I think mental health is really big and being brought up within COVID… not having sound support through education… there's not enough support. We're burning out. We're overworking, we can't focus on studies and we're becoming stressed. *(Participant # 7)*.

The rising trends in vaping amongst young people were also flagged as troubling. Pressure from peers and media trends, uncertainty around its impact on health and appealing and seemingly innocuous flavours of vapes were thought to be fuelling the vaping epidemic.

The reason people vape more than smoke is that you can smell when someone has smoked (cigarettes), and vaping is not like that. *(Participant #9)*.

Interview participants differed in their views on responsibility, with some feeling that some young adults make ‘poor lifestyle choices’; others pointed out that young people are committed to staying healthy, but broader social forces, such as societal norms, peer pressure and social media advertisements and promotion, constrain choices.

For the younger generations…we are pretty healthy and understand what it is to be healthy rather than just dieting and losing weight. *(Participant # 8)*.

100% the Internet contributes to the like alcohol and vape presence in Australia at the moment. *(Participant # 4)*.

Social expectations and norms of ‘Australian behaviours’ and learnt behaviours from youth were mentioned as heavy influencers of choices, especially in terms of alcohol use.

Australia's kind of normalised drinking alcohol… so drinking responsibly, I think, is a bit of an issue. *(Participant # 7)*.

People drink socially or grew up drinking socially and then sort of just continue that into their adult life. *(Participant # 6)*.

The high cost of living was a recurring and cross‐cutting theme across interviews. Participants described how financial pressures constrained their ability to eat healthy and to stay active despite being well aware of the importance of these behaviours for overall health and well‐being. Young adults in the current study expressed their desires to eat healthy and stay active, and positioned these things as difficult to achieve, rather than undesirable. Financial strain limited their opportunities for positive work‐life balance and access to services, thus negatively impacting their mental well‐being and physical health. This evoked a sense of hopelessness amongst participants as it felt like a trouble out of their own control, but rather a deep societal issue. Furthermore, major global crises with financial impacts further exacerbated existing anxieties by disrupting social life and support systems.

The cost of things nowadays… like eating healthy… buying chocolate is so much cheaper than spinach, so obviously I'm going to get that. And going to the gym is so expensive. *(Participant # 4)*.

A big impact for young adults is their family life, which is something they can't really control and that can hugely affect health, both physically and mentally because if your family is really poor you might not be able to afford the same food as other people, which means you don't grow in the same way. *(Participant # 12)*.

### 3.2 Reflections on Responses to Concerns

A key recommendation that arose during discussions was to prioritise young adults in research and service design that focuses on providing mental health support for the impacts of the economic crisis they have to navigate.

Participants also emphasised the importance of introducing awareness campaigns early in schooling years as a part of programmatic responses. The nature of the recommended responses closely mirrored beliefs around root causes and key drivers of concerns. For example, the perceived lack of clear information on the risks associated with vaping highlighted the need for targeted education campaigns. Emphasis was also placed on including younger adolescents in these prevention efforts across multiple interviews; as these young people suggest, educational institutions such as schools are ideal platforms for health promotion efforts.

Schools [are] definitely the place for teaching young adults [about vaping]. I feel like, the earlier the better. *(Participant # 4)*.

Early mental health interventions, which I think should be introduced in school… educating about what's should be avoided, but also what you can do and should do to make yourself feel better. *(Participant # 12)*.

Participants varied in their views on styles of campaigns, with one suggesting fear‐based approaches would be most effective.

Go around to schools and talk about things that are bad for you and… because there are, especially a lot of young boys, are focusing on their health now, they wanna bulk for the gym and they wanna do this kind of thing so I guess scaring them out of vaping and putting that money towards gym. *(Participant # 8)*.

Others felt more conventional methods—websites, school‐based counselling support—would be helpful. Creating supportive networks through the establishment of community groups, targeted family support and even sports‐based programmes were also identified as ideas for programmatic responses. Some participants were in favour of enacting bans on vapes.

Similar responses were recommended in relation to mental health concerns such as eating disorders. Information to help with recognising and managing eating disorders, with a greater in‐school emphasis on developing a healthy relationship with food and exercise, was suggested.

I guess better promoting in schools. I think even with nutrition and stuff, I think schools don't do a very good job of promoting healthy nutrition and a healthy diet in a way that's not mentally detrimental. *(Participant #5)*.

In addition to information campaigns, some participants acknowledged the need for responses to address the barriers to accessing support, such as costs. Suggestions to leverage schools as a platform to provide free, easy‐to‐access mental health support were a response that particularly resonated.

Mental health interventions, which I think should be introduced in schools for free and early education about things, so… educating about what should be avoided, but also what you can do, and should do, to make yourself feel better. *(Participant # 12)*.

Most agreed that economic burdens were not an issue that a specific programme could assist with, but rather a problem that governments needed to address with specific strategies and plans. Inadequate government funding and low minimum wages were described as adding further pressure caused by the prohibitively high cost of living. The call for a ‘government response’, through policy reform and additional financial support services, reflects the view that action on structural determinants is required, as there are things that are out of young people's control.

A lot of counselling isn't covered by Medicare and even to get a [mental] health plan is expensive… it's not as accessible as it could be. *(Participant # 4)*.

### 3.3 Involvement in Response Formulation and Expectations Around Engagement

The common theme from interviews was enthusiasm for more meaningful engagement in co‐designing responses to key health needs. The majority were motivated by the desire to contribute to efforts to keep themselves and their peers happy and healthy. The value of first‐hand experience was emphasised as being critical to ensuring programmatic responses are successful in meeting young adult needs, with most young adults agreeing that their experiences are unique to their own age group and one that cannot be fully understood and assumed by older generations. However, hardly anyone reported being offered an opportunity to be involved in the conceptualisation or design of programmatic responses to improve young adult health.

Everything is so different from even 10 years ago… the stakeholders and the people who are creating all these programmes, who are 50 years older than me, let alone the 60–70 years older than the young people, just don't know. We need to be discussing it with them [young people]. What do they actually want? Don't make decisions for them just because they're young people, because you're going to get such backlash, and you're putting so much money into something that isn't going to work. *(Participant # 8*).

I want to make a change, and I want to help people reach their best potential because I've been fortunate enough to have a pretty good life and pretty good upbringing, so I feel like I have a really good advantage in the world, but a lot of people are really disadvantaged and don't have that. And that's not fair. *(Participant # 7)*.

I've struggled with some health issues in my time, so like it's not a good feeling in yourself. It's not great, so just making sure that everyone has a good time with themselves and knows what's good for themselves. *(Participant # 3)*.

Reflecting on expectations around involvement, young people spoke of ensuring that alignment between tasks, interests and skills. Considerations around time commitments and remuneration were also touched on by most of the participants, enabling all young adults to participate in co‐design programmes without additional financial or time burdens.

I'd like to do an education and promotion kind of programme, or like going to a talk or something, probably. I probably wouldn't want to be part of like an education programme where I would have to educate and talk to teenagers or anything though, because I hate talking in front of people. *(Participant # 6)*.

Ensuring that participants were adequately reimbursed for their time and contributions was highlighted as an important aspect of participation.

Unfortunately, there's just not enough time in the day to be giving up paid hours anymore. *(Participant # 2)*.

One participant mentioned her anxieties working with other young adults on issues they feel passionate about but do not participate in, using the example of vaping to illustrate her worries of not being able to connect to the audience.

I don't vape but I want to help the people that do vape, and they'll just look at me like I'm a loser, you know what I mean. *(Participant # 4)*.

Using platforms for engagement that suit young people's preferences was mentioned as an important consideration for programme participation. For one young woman, this meant being able to engage in co‐design in person rather than online.

If being involved in that kind of programme meant Zoom meetings and sitting down at my desk online on my computer by myself, I would hate it; if it were an interactive in‐person group meeting, then I would love it. *(Participant # 4)*.

## 4 Discussion

In this qualitative study, we explored the views of young adults on priority health issues and their involvement in shaping health promotion responses. Mental health appeared as a major health concern amongst young adults. Key barriers to health and wellbeing included limited awareness of the risks associated with vaping and eating disorders, peer pressure to vape, financial strain and cost‐of‐living pressures that restrict access to services and healthier lifestyle choices. Response emphasised the need for interventions to be aligned with key determinants, such as education and awareness. Early intervention, specifically in schools, was mentioned by many as a key way to begin prevention efforts early for all young people. Participants also suggested free, accessible support services and programmes to increase access to essential care and education. These responses underscored the need for structural changes, including policy‐level responses from government, to create an enabling environment for sustained impact. Lived experience was emphasised as pivotal for ensuring that the design and planning of interventions were relevant to young people and responsive to their needs. Participants shared their interest in opportunities aligned with their interests, skills, availability and engagement preferences, but reported limited opportunities to co‐design interventions.

The study highlighted mental health concerns (e.g., anxiety, bullying and eating disorders) and vaping as important concerns for young adults living in South Australia. These concerns are reflected in data from the Australian Institute of Health and Welfare (AIHW), reporting mental health and substance use disorders as the major contributors to the high burden of disease amongst young people [4]. Cost of living and financial constraints were also emphasised as key determinants in the current study, commonly minimising opportunities to stay healthy and access supports. Reflecting these findings, the 2024 Youth Survey also reported cost of living and mental health as the important issues recognised by young people in Australia [6]. These similar findings suggest an urgent need for action to ease the financial burdens on young adults, thereby allowing them equal opportunities to live balanced lifestyles and to access medical and mental health supports when needed.

Social norms at both the peer and societal levels influence an individual's ability to make healthier choices [19, 20]. Studies suggest both social support and social norms, often established through social media and mass campaigns, predict health behaviours [19, 20]. Social norm campaigns, in general and social media, have proven effective in multiple health promotion settings, including anti‐smoking [21], traffic safety and protective measures during the COVID‐19 pandemic [22]. Young adults in the study who observed vaping and drinking behaviours reported initiation of these behaviours due to peer pressure and pressures from societal norms. This aligns with existing research indicating that young people perceive marketing campaigns, particularly on social media, as having a substantial impact on their use of harmful products, such as vapes [15, 23]. Further research indicates that young Australians perceive social media marketing of harmful products like gambling and vaping as having a strong influence on their usage [15, 23]. This study aligns with previous findings, highlighting the powerful role social norms play in shaping young adults' behaviours and perceptions [24, 25]. Consequently, health promotion efforts should utilise social media campaigns aimed at Australian youth, employing targeted marketing and co‐design recruitment incentives.

Further, the study recorded extensive interest of young adults in co‐designing responses to key health concerns; however, there were limited opportunities offered. A scoping review by Sellars et al. also reports on the low level of engagement of young people in health research [26]. It's important to note that the involvement of young people in the design and planning of health research is gradually growing as the field expands [27, 28]. However, significant barriers continue to exist at the research, organisation and funding levels, including a lack of resources and funding, challenges in accessing young people and complex ethical approvals [29]. These barriers often prevent young people from engaging with researchers in designing health incentives, with few opportunities provided for them despite their openness to participation [9, 29]. Organisations and funding bodies must, therefore, work to increase their engagement with young adults who wish to be heard and represented in health incentives. Establishing youth advisory groups, integrating co‐design approaches into programme development, and partnering with youth‐centred institutions could be integrated into organisations to ensure youth‐led initiatives are achieved. Further, funding bodies and policy should allocate dedicated grants and funding opportunities for youth‐led initiatives, ensuring the participants are well supported and empowered.

Young people had clear expectations for engagement. These included ensuring that interests, skills and engagement platforms aligned with preferences, and that connections could be built amongst all young people involved. Considerations around their availability and ensuring appropriate remuneration for their time and contributions were also flagged as important. Young adults expressed willingness to join a programme if it were freely available, aligned with their area of interest and involved familiar individuals. These findings align with the existing research emphasising appropriate reimbursements for time and contribution of young people invested in the decision‐making initiatives [14, 30]. Research additionally shows the importance of building strong, trusting relationships between young adult participants and key stakeholders to further incentivise meaningful engagement [29]. Withstanding collaborative research must ensure the interests, skills and engagement platforms align with the preferences of the young adults participating [29, 30]. Therefore, it is vital that young adults receive transparent information about their engagement and are appropriately reimbursed for their valuable insights.

Leveraging these findings in health promotion practise can support the development of youth‐focused initiatives that are meaningful, relevant and responsive to the needs of young Australians. Co‐designing with young adults ensures that messaging, delivery methods and priorities align with their lived experiences, preferences and communication styles. This collaborative approach can enhance the relatability of health messages, thus increasing the likelihood that they will be understood, accepted and acted upon by young people.

### 4.1 Limitations

Whilst this study provides valuable insights into the health priorities of adolescents in South Australia, it is important to note that our study has some limitations. The sample included mostly females in their early 20's who were well‐educated and thus may not represent the experiences and views of diverse genders and ages in SA. Further, whilst our sample was culturally diverse, the small sample size limited the diverse perspectives of many important young adults in SA, including those without tertiary education and from non‐English speaking backgrounds. Additionally, most of our study came from urban settings; thus, those living in rural areas were not represented in the study outcomes. Therefore, the participants in the current study are likely to share common concerns, and there may be additional health concerns unique to different groups not captured here.

## 5 Conclusion

This study amplifies the voices of young adults in South Australia, revealing a pressing need to address mental health challenges, the rising prevalence of vaping, deeply influenced by social norms and media campaigns, lack of knowledge, peer pressure, financial constraints and systematic barriers to care. Despite their willingness and enthusiasm to engage, young people in this study expressed that they face limited opportunities to shape the health interventions meant for them. Effective and sustainable solutions must not only align with the lives and realities of young adults but also actively involve them in the design, implementation and evaluation of programmes and policies.

We call on governments, health systems, researchers, policy makers and educational institutions to prioritise early, school‐based interventions, ensure universal access to affordable and youth‐friendly mental health and substance use services, and implement structural policies that mitigate the rising cost of living and its adverse effects on health. More importantly, we urge stakeholders to create meaningful and resourced pathways for young people to co‐design solutions, with appropriate remuneration, flexibility and respect for their lived expertise. Empowering young people is not just the right thing to do; it is also key to creating health systems and communities that are fair, inclusive and prepared for the future.

## Author Contributions

Z.S.L., J.K. and S.M. conceptualised the study. Z.A.P. and Z.S.L. developed the study questionnaire and received feedback from all authors. Interviews were conducted, transcribed and analysed by P.C. and Z.A.P. and reviewed by Z.S.L. The first draft of the manuscript was written by P.C. and Z.A.P. All authors reviewed and approved the manuscript.

## Funding

This study was funded by the University of Adelaide internal grant scheme, i.e., Robinson Research Institute Engaging Opportunity Grant 2022. Z.S.L. (# GNT2009730) is an NHMRC Investigator Fellow.

## Ethics Statement

Ethical approval was obtained from the Human Research Ethics Committee of the University of Adelaide (H‐2023‐157). At the time of recruitment, participants were provided with a participant information sheet outlining what was involved in participation as well as the risks and benefits of participation to support informed decision‐making.

## Consent

Written consent was obtained from young adults wishing to take part in the study.

## Conflicts of Interest

The authors declare no conflicts of interest.

## Supporting information


**File S1:** Checklist‐standards for reporting qualitative research (SRQR).

**File S2:** hpja70202‐sup‐0002‐Supplementary_File_S2.docx.

## Data Availability

The data that support the findings of this study are openly available in Figshare at https://doi.org/10.25909/29622071.
