# Supplementary material for: Young Adults' Views on Priority Health Issues and Their Involvement in Shaping Responses: A Qualitative Exploration in South Australia
Source: Health Promot J Austr. 2026 Jun 5;37(3):e70202. doi: 10.1002/hpja.70202 (PMC13238411; doi:10.1002/hpja.70202)
Supplement: Supplementary file 2 — File S2: hpja70202‐sup‐0002‐Supplementary_File_S2.docx. [file HPJA-37-0-s002.docx]

**All for One: Exploring Strategies to Enhance Stakeholder Involvement in Participatory Research to Maximize Gains in Adolescent and Young Adults’ Health**

**Interview guide for adolescents and young adults**

1. What do you understand by the term adolescents and young adults?
2. What does health and wellbeing mean to you? What are some things that you do to be healthy?
3. How do you feel about the health and wellbeing of the general population? And the population of the young people around you?
   1. What are the main health concerns and risk for young people?
4. In your opinion, what could be some helpful interventions to improve the health of adolescents and young adults? What would these look like and how would they be promoted and function?
5. From where do you get information about the health of adolescents and young adults?
6. Have you or your friend or a closed one been part of any outreach/school program which involved adolescents and young adults? If yes, ask if that program was to improve the health of adolescents and young adults.
7. Are you aware of some previous or current government or community-based programs to improve the health of adolescents and young adults?
   1. **If yes,** can you tell us anything about that program? What do they intend to do? What are they aiming to change?
8. In your opinion, the involvement and engagement of adolescents and young adults in the design of health and research programs could help improve adolescent and young adults' health outcomes.
   1. **If yes,** ask why they think it's beneficial and how they believe that adolescent engagement can help maximise impact on adolescent and young adults’ health.
9. Have you or your close friend or relatives ever been apart of any health program which engaged adolescents and young adults in the planning and designing phase of a program?
   1. Ask the details of the program and ask how the program was different from the other programs which did not involve adolescents/participants.
10. If given an opportunity, would you like to engage and get involved in developing health research programs to improve the health outcomes of adolescents and young adults?
    1. **If yes,** ask what you would like to do. How would it improve the lives of people? What are their skills which would benefit this work?
11. What interests you in working for better health and well-being of adolescents and young adults?
12. Is there anything you would like to share or ask us?
